# Effectiveness of pneumococcal polysaccharide vaccine for preschool-age children with chronic disease.

**DOI:** 10.3201/eid0506.990616

**Published:** 1999

**Authors:** A E Fiore, O S Levine, J A Elliott, R R Facklam, J C Butler

**Affiliations:** Centers for Disease Control and Prevention, Atlanta, Georgia 30333, USA. abf4@cdc.gov

## Abstract

To estimate the effectiveness of pneumococcal polysaccharide vaccine, we serotyped isolates submitted to the Pneumococcal Sentinel Surveillance System from 1984 to 1996 from 48 vaccinated and 125 unvaccinated children 2 to 5 years of age. Effectiveness against invasive disease caused by serotypes included in the vaccine was 63%. Effectiveness against serotypes in the polysaccharide vaccine but not in a proposed seven-valent protein conjugate vaccine was 94%.


### Effectiveness of Pneumococcal Polysaccharide Vaccine for Preschool-Age Children with Chronic Disease

**Anthony E. Fiore, Orin S. Levine, John A. Elliott, Richard R. Facklam, Jay C. Butler, for the Pneumococcal Sentinel Surveillance Working Group**
Centers for Disease Control and Prevention, Atlanta, Georgia, USA

To estimate the effectiveness of pneumococcal polysaccharide vaccine, we serotyped isolates submitted to the Pneumococcal Sentinel Surveillance System from 1984 to 1996 from 48 vaccinated and 125 unvaccinated children 2 to 5 years of age. Effectiveness against invasive disease caused by serotypes included in the vaccine was 63%. Effectiveness against serotypes in the polysaccharide vaccine but not in a proposed seven-valent protein conjugate vaccine was 94%.

*Streptococcus pneumoniae* is a leading cause of pneumonia, meningitis, bacteremia, and death in young children. A polysaccharide vaccine has been recommended for use in chronically ill children and adults 2 to 64 years of age, as well as all adults ≥65 (1). While many studies have assessed the immunogenicity of the polysaccharide vaccine, scant data exist on its effectiveness in younger children.

More than 90 serotypes of *S. pneumoniae* have been described (2); however, most invasive infections in the United States are caused by ≤10 serotypes (3). Pneumococcal vaccines available since 1978 consist of a mixture of capsular polysaccharides from the most common serotypes causing invasive disease. This vaccine is recommended for children ≥2 years of age with underlying diseases or immunosuppressive medical treatments that are risk factors for invasive pneumococcal disease (1,3).

Clinical trials of pneumococcal polysaccharide vaccine effectiveness in children have shown conflicting results (4-7). Vaccine failure in immunized children has been reported (8), and one study comparing immunization with antibiotic prophylaxis in children with sickle cell disease concluded that the vaccine was inferior to penicillin prophylaxis (9). Uncertainty regarding the effectiveness of vaccination may contribute to low vaccination rates among persons at risk for pneumococcal disease (1).

In indirect cohort analysis (10), the distribution of pneumococcal serotypes causing invasive disease among vaccinated and unvaccinated groups is compared. If the vaccine is effective, vaccinated persons have fewer infections with serotypes represented in the vaccine than unvaccinated persons. This method has been used to calculate an overall effectiveness of 57% in persons >5 years of age, based on serotypes of invasive isolates obtained through a national, voluntary sentinel surveillance system (11). Using data from national surveillance, we examined vaccine effectiveness for children 2 through 5 years of age.

### The Study

Since 1978, a national, hospital laboratory-based surveillance system has collected data on invasive pneumococcal disease (12). Participating institutions are requested to report all pneumococcal isolates obtained from normally sterile body sites, along with information on the patient's age, sex, symptoms, underlying diseases, and vaccination history. The specifics of how demographic and vaccination information is collected are the responsibility of participating institutions. Isolates are serotyped at the

Address for correspondence: Anthony E. Fiore, National Center for Infectious Diseases, Centers for Disease Control and Prevention; 1600 Clifton Road, Mail Stop G37, Atlanta, GA 30333, USA; fax: 404-639-1538; e-mail: abf4@cdc.gov.

Centers for Disease Control and Prevention on the basis of capsular swelling with serotype-specific antisera (Quellung reaction).

Children included in the analysis were 24 to 59 months of age with one or more chronic illnesses, had vaccination status and date indicated on the surveillance form, received vaccine between January 1984 and April 1996, and had onset of invasive pneumococcal disease between January 1984 and April 1996. Only isolates from cerebrospinal fluid (CSF) or blood were considered in the analysis. Information on antibiotic prophylaxis was not collected. Chronic illness was defined as an underlying illness considered a risk factor for invasive pneumococcal disease and an indication for vaccination (3).

Vaccine effectiveness was defined as the percentage of reduction in the risk for infection from serotypes included in the vaccine (vaccine-type serotypes) among vaccinated persons compared with unvaccinated persons. Infections with vaccine-related serotypes (6A, 9A, 9L, 18B, 18F, 23A) not specifically included in the vaccine were categorized as infections with nonvaccine serotypes, except where noted. Effectiveness was expressed as 1 minus the odds ratio x 100%; the 95% confidence intervals (also x 100%) were calculated by the methods of Cornfield when cell sizes were all greater than five subjects and by exact methods otherwise. Calculations were performed with Epi-Info version 6.02 (CDC/World Health Organization, Atlanta, GA) with the EXACT supplemental program (David O. Martin).

We performed a preliminary analysis of all pneumococcal isolates from children in the database to determine the proportion of vaccine-type organisms in unvaccinated persons by sex, underlying illness, or state of residence. Proportions of vaccine-type serogroups did not differ by underlying illness or by sex. Because the proportion of vaccine-type isolates from children from Alaska was 92.3%, compared with the 85.4% of isolates from children from other states (chi-square = 6.3; p <0.02), children from Alaska were excluded from the analysis.

The analysis included 173 children, 52% male, median age 3 years; 48 children (28%) had received vaccine before acquiring invasive pneumococcal disease. Isolates were obtained from blood only from 156 children (90%), from CSF only from 10 children (6%), and from both sites from 7 children (4%). The median time between date of vaccination and date of specimen collection was 338.5 days (33 days to 1,341 days), and no child had been vaccinated within 30 days of invasive pneumococcal infection. Of serotypes from the 173 invasive infections, serotypes 4, 6A, 6B, 14, 23F, 19F, 9V, and 18C accounted for 81% of the isolates (Figure 1).

Forty-six (27%) of children in the study had sickle cell disease (Figure 2). The "other" category included children with congenital anomalies such as congenital heart or lung defects, children with anatomic asplenia, and children on immunosuppressive medication regimens. Thirty-three (69%) of 48 vaccinated children had sickle cell disease.

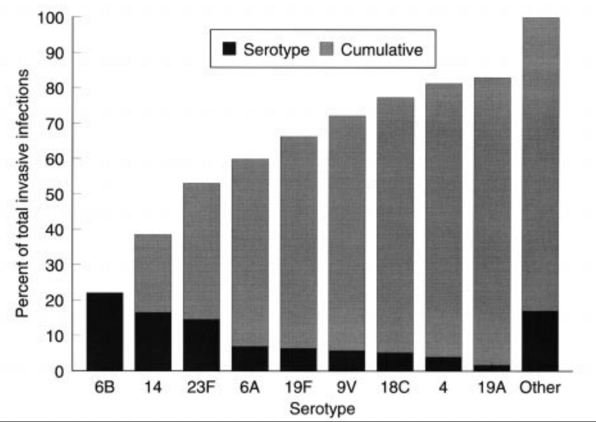

Figure 1. Invasive pneumococcal infections among 173 children ages 2 through 5 years (24-59 months), by serotype. Bottom bar represents proportion of total invasive infections in the cohort caused by each serotype. Top bar depicts cumulative proportion of invasive infections caused by serotypes represented by the bars to the left. Serotypes in the "other" category included 19 serotypes with three or fewer isolates. Two isolates could not be serotyped.

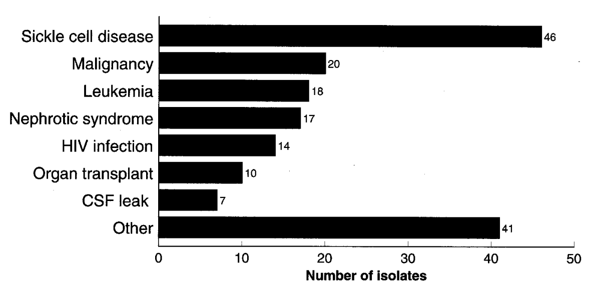

Figure 2. Frequency of various underlying chronic illnesses among 173 children with invasive pneumococcal disease. The category "malignancy" excluded hematopoetic malignancies, which are included in the leukemia category. Organ transplant includes both solid organ and bone marrow transplants. CSF is cerebrospinal fluid.

The Table presents vaccine effectiveness estimates for the overall cohort and for children with and without sickle cell disease. For children with the disease, the lower bound of the 95% confidence interval included 0%. The estimate of vaccine effectiveness for children without sickle cell disease was higher than the estimate for children with the disease. Point estimates of effectiveness for children with nephrotic syndrome or HIV infection were 80%; however, the 95% confidence intervals included 0% (data not shown). Other chronic diseases reported in this cohort included leukemia, nonhematopoetic malignancy, and organ transplant; however, none of the children with these underlying diseases were vaccinated, and effectiveness could not be calculated.

Protein conjugate vaccines offer the advantage of being effective in the first 2 years of life, when response to polysaccharide vaccines is poor. However, the number of serotypes that can be represented in these vaccines is limited. To evaluate polysaccharide effectiveness for serotypes not represented in a protein conjugate vaccine under evaluation for license (13), we excluded children infected with serotypes 4, 6B, 9V, 14, 18C, 19F, and 23F. Polysaccharide vaccine was highly effective in preventing invasive disease due to serotypes included in the polysaccharide vaccine but not in the conjugate vaccine (Table). If the 14 children with serotypes 6A, 9A, 9L, 18B, 18F, and 23A are also excluded (because of potential protection conferred by the proposed conjugate vaccine for these vaccine-related serotypes), the vaccine effectiveness estimate is 92% (exact 95% confidence intervals 17% to 100%).

### Conclusions

Case-control studies have demonstrated that pneumococcal capsular polysaccharide vaccines are effective (14-16) and cost-effective (17,18) in the prevention of invasive pneumococcal disease among elderly and chronically ill adults. We used data from a national sentinel surveillance system for invasive pneumococcal disease to determine whether children ages 2 to 5 years were also protected. An overall vaccine effectiveness of 63% was demonstrated by indirect cohort analysis (15). The indirect cohort analysis presented here strengthens the case for the use of pneumococcal polysaccharide vaccine for children with underlying conditions. For children with sickle cell disease, penicillin prophylaxis remains the most effective preventive measure for reducing pneumococcal disease.

Accuracy of vaccine history is critical to this analysis and may vary between surveillance sites. To minimize inaccuracies, patients with no indication of vaccine history were excluded. For those with a reported vaccine history, misclassification due to inaccurate history should be as likely among patients with vaccine-type as among nonvaccine-type infections because the serotype of patient isolates was not known when vaccine status was determined (serotyping was done at CDC). Bias due to this nondifferential misclassification will be towards the null hypothesis (no effect of vaccination) (19).

Newly developed pneumococcal protein conjugate vaccines are safe and immunogenic for infants and young children (13,20,21). Preliminary results from a large, Phase-III trial of a heptavalent conjugate vaccine among healthy children indicate substantial efficacy in preventing invasive disease (13). However, the expense and technical difficulty of creating conjugates for each serotype will likely limit the number of serotypes represented in a polyvalent conjugate vaccine to fewer than 12. Available data suggest that polysaccharide vaccine, when administered after primary immunization with a conjugate vaccine, elicits a significant booster effect in

Table. Estimates of pneumococcal polysaccharide vaccine effectiveness among 173 children 2 through 5 years of age, using the indirect cohort method

| | Vaccine serotype/total(%) | | |
|---|---|---|---|
| Group | Vaccinated children[a] | Unvaccinated children[a] | Effectiveness (95% CI)[b] |
| All children | 35/48 (73) | 110/125 (88) | 63% (8% to 85%) |
| Children with SCD | 27/33 (82) | 12/13 (92) | 62% (-294% to 98%) |
| Children without SCD | 8/15 (53) | 98/112 (88) | 84% (40% to 96%) |
| Nonconjugate vaccine serotype[c] | 1/14 (7) | 18/33 (55) | 93% (45% to 100%) |

[a]23-valent pneumococcal polysaccharide vaccine.
[b]Effectiveness (95% confidence interval) estimated as (1- odds ratio or 95% confidence bound) x 100%.
[c]Children infected with a serotype not in proposed conjugate vaccine (15) (excludes children infected with serotypes 4, 6B, 9V, 14, 18C, 19F, 23F).
SCD, sickle-cell disease.

healthy infants (22) equivalent to the booster response engendered by a second conjugate vaccine series (23). These results and the level of effectiveness seen with pneumococcal polysaccharide vaccine in our study suggest that the polysaccharide vaccine will still be a useful adjunct to conjugate vaccine, by providing additional protection to children ≥2 years of age for whom polysaccharide vaccine is currently indicated.

### Acknowledgments

The authors thank A.R. Franklin, D. Jackson, L. LaClaire, and N. Pigott for serotyping of pneumococcal isolates and the members of the Pneumococcal Sentinel Surveillance Working Group: Carol Camp, Patricia Charache, Mel Jackson, W. Keith Hadley, Joan Hoppe-Bauer, Michael R. Jacobs, Phyllis Tyler, Janet Monahan, Harold Moore, Jane D. Siegel, David Sherer, and David Welch.

Dr. Fiore is a medical epidemiologist in the Hepatitis Branch, Division of Viral and Rickettsial Diseases, National Center for Infectious Diseases.

### References

1. Centers for Disease Control and Prevention. Prevention of pneumococcal disease: recommendations of the Advisory Committee on Immunization Practices (ACIP). MMWR Morb Mortal Wkly Rep 1997;46(RR-8).
2. Henrichsen J. Six newly recognized types of *Streptococcus pneumoniae*. J Clin Microbiol 1995;33:2759-62.
3. American Academy of Pediatrics. Pneumococcal infections. In: Peter G, editor. 1997 Red book: report of the Committee on Infectious Diseases. 24th ed. Elk Grove Village (IL): American Academy of Pediatrics; 1997. p. 410-9.
4. Riley ID, Everingham FA, Smith DE, Douglas RM. Immunisation with a polyvalent pneumococcal vaccine: Effect on respiratory mortality in children living in the New Guinea highlands. Arch Dis Child 1981;56:354-7.
5. Rosén C, Christensen P, Hovelius B, Prellner K. Effect of pneumococcal vaccination on upper respiratory tract infections in children: Design of a follow-up study. Scand J Infect Dis 1983;Suppl 39:39-44.
6. Douglas RM, Miles HB. Vaccination against *Streptococcus pneumoniae* in childhood: lack of demonstrable benefit in young Australian children. J Infect Dis 1984;149:861-9.
7. Ammann AJ, Addiego J, Wara DW, Lubin B, Smith WB, Mentzer WC. Polyvalent pneumococcal-polysaccharide immunization of patients with sickle-cell anemia and patients with splenectomy. N Engl J Med 1977;297:897-900.
8. Ahonkhai VI, Landesman SH, Fikrig SM, Smalzer EA, Brown AK, Cherubin CE, et al. Failure of pneumococcal vaccine in children with sickle-cell disease. N Engl J Med 1979;301:26-7.
9. John AB, Ramlal A, Jackson H, Maude GH, Waight-Sharma, A, Serjeant GR. Prevention of pneumococcal infection in children with homozygous sickle cell disease. BMJ 1984;288:1567-70.
10. Broome CV, Facklam RR, Fraser DW. Pneumococcal disease after pneumococcal vaccination: an alternative method to estimate the efficacy of pneumococcal vaccine. N Engl J Med 1980;549-52.
11. Butler JC, Breiman RF, Campbell JF, Lipman HB, Broome CV, Facklam RR. Pneumococcal vaccine efficacy: an evaluation of current recommendations. JAMA 1993;270:1826-31.
12. Broome CV. Efficacy of pneumococcal polysaccharide vaccines. Rev Infect Dis 1981;Suppl 3:S82-8.
13. Black SB, Shinefield H, Ray P, Lewis E, Fireman P, et al. Efficacy of heptavalent conjugate pneumococcal vaccine (Wyeth Lederle) in 37,000 infants and children: results of the Northern California Kaiser Permanente Efficacy Trial. In: Programs and abstracts of the 38th Interscience Conference on Antimicrobial Agents and Chemotherapy; 1998; San Diego, California. Washington: American Society for Microbiology, 1998.
14. Shapiro ED, Berg AT, Austrian R, Schroeder D, Parcells V, Margolis A, et al. The protective efficacy of polyvalent pneumococcal polysaccharide vaccine. N Engl J Med 1991;325:1453-60.
15. Sims RV, Steinmann WC, McConville JH, King LR, Zwick WC, Schwartz JS. The clinical effectiveness of pneumococcal vaccine in the elderly. Ann Intern Med 1988;108:653-7.
16. Farr BM, Johnston BL, Cobb JK, Fisch MJ, Germanson TP, Adal KA, et al. Preventing pneumococcal bacteremia in patients at risk: Results of a matched case-control study. Arch Intern Med 1995;155:2336-40.
17. Gable CB, Holzer SS, Engelhart L, Friedman RB, Smeltz F, Schroeder D, et al. Pneumococcal vaccine: efficacy and associated cost savings. JAMA 1990;264:2910-5.
18. Sisk JE, Moskowitz AJ, Whang W, Lin JD, Fedson DS, McBean AM, et al. Cost-effectiveness of vaccination against pneumococcal bacteremia among elderly people. JAMA 1997;278;1333-9.
19. Copeland KT, Checkoway H, Holbrook RH, McMichael AJ. Bias due to misclassification in the estimate of relative risk. Am J Epidemiol 1977;105:488-95.
20. Käyhty H, Åhman H, Rönnberg P-R, Tillikainen R, Eskola J. Pneumococcal polysaccharide-meningococcal outer membrane protein complex conjugate vaccine is immunogenic in infants and children. J Infect Dis 1995;172:1273-8.
21. Rennels MB, Edwards KM, Keyserling HL, Reisinger KS, Hogerman DA, Madore DV, et al. Safety and immunogenicity of heptavalent pneumococcal vaccine conjugated to $CRM_{197}$ in United States infants. Paediatrics 1998;101:604-11.
22. Åhman H, Käyhty H, Lehtonen H, Leroy O, Froeschle J, Eskola J. *Streptococcus pneumoniae* capsular polysaccharide-diphtheria toxoid conjugate vaccine is immunogenic in early infancy and able to produce immunologic memory. Pediatr Infect Dis J 1998;17:211-6.
23. Obaro SK, Huo Z, Banya WAS, Henderson DC, Monteil MA, Leach A, et al. A glycoprotein conjugate vaccine primes for antibody responses to a pneumococcal polysaccharide vaccine in Gambian children. Pediatr Infect Dis J 1997;16:1135-40.

